# Preoperative Identification of Medullary Thyroid Carcinoma (MTC): Clinical Validation of the Afirma MTC RNA-Sequencing Classifier

**DOI:** 10.1089/thy.2022.0189

**Published:** 2022-09-14

**Authors:** Gregory W. Randolph, Julie Ann Sosa, Yangyang Hao, Trevor E. Angell, David C. Shonka, Virginia A. LiVolsi, Paul W. Ladenson, Thomas C. Blevins, Quan-Yang Duh, Ronald Ghossein, Mack Harrell, Kepal Narendra Patel, Michael H. Shanik, S. Thomas Traweek, P. Sean Walsh, Michael W. Yeh, Amr H. Abdelhamid Ahmed, Allen S. Ho, Richard J. Wong, Joshua P. Klopper, Jing Huang, Giulia C. Kennedy, Richard T. Kloos, Peter M. Sadow

**Affiliations:** ^1^ Division of Thyroid and Parathyroid Endocrine Surgery, Department of Otolaryngology-Head and Neck Surgery, Massachusetts Eye and Ear, Harvard Medical School, Boston, Massachusetts, USA.; ^2^ Department of Surgery, Massachusetts General Hospital, Harvard Medical School, Boston, Massachusetts, USA.; ^3^ Department of Surgery, University of California San Francisco (UCSF), San Francisco, California, USA.; ^4^ Department of Research and Development, Veracyte, Inc., South San Francisco, California, USA.; ^5^ Division of Endocrinology and Diabetes, Keck School of Medicine, University of Southern California, Los Angeles, California, USA.; ^6^ Department of Otolaryngology—Head and Neck Surgery, University of Virginia, Charlottesville, Virginia, USA.; ^7^ Anatomic Pathology Division, Department of Pathology and Laboratory Medicine, University of Pennsylvania School of Medicine, Philadelphia, Pennsylvania, USA.; ^8^ Division of Endocrinology, Diabetes and Metabolism, Department of Medicine, Johns Hopkins University, Baltimore, Maryland, USA.; ^9^ Texas Diabetes and Endocrinology, Austin, Texas, USA.; ^10^ Section of Endocrine Surgery, Department of Surgery, University of California San Francisco, San Francisco, California, USA.; ^11^ Department of Pathology and Laboratory Medicine, Memorial Sloan-Kettering Cancer Center, New York, New York, USA.; ^12^ The Memorial Center for Integrative Endocrine Surgery, Hollywood, Weston and Boca Raton, Florida, USA.; ^13^ Division of Endocrine Surgery, Department of Surgery, NYU Langone Medical Center, New York, New York, USA.; ^14^ Endocrine Associates of Long Island, Smithtown, New York, USA.; ^15^ Thyroid Cytopathology Partners, Austin, Texas, USA.; ^16^ Section of Endocrine Surgery, UCLA David Geffen School of Medicine, Los Angeles, California, USA.; ^17^ Department of Otolaryngology, Cedars-Sinai Medical Center, Los Angeles, California, USA.; ^18^ Head and Neck Service, Department of Surgery, Memorial Sloan Kettering Cancer Center, New York, New York, USA.; ^19^ Department of Medical Affairs, Veracyte, Inc., South San Francisco, California, USA.; ^20^ Department of Pathology, Massachusetts General Hospital and Harvard Medical School, Boston, Massachusetts, USA.; ^21^ Department of Otolaryngology-Head and Neck Surgery, Harvard Medical School, Massachusetts Eye and Ear, Boston, Massachusetts, USA.

**Keywords:** indeterminate cytology, machine learning, medullary thyroid cancer, molecular diagnostics, molecular testing, thyroid nodule

## Abstract

**Background:**

Cytopathological evaluation of thyroid fine-needle aspiration biopsy (FNAB) specimens can fail to raise preoperative suspicion of medullary thyroid carcinoma (MTC). The Afirma RNA-sequencing MTC classifier identifies MTC among FNA samples that are cytologically indeterminate, suspicious, or malignant (Bethesda categories III–VI). In this study we report the development and clinical performance of this MTC classifier.

**Methods:**

Algorithm training was performed with a set of 483 FNAB specimens (21 MTC and 462 non-MTC). A support vector machine classifier was developed using 108 differentially expressed genes, which includes the 5 genes in the prior Afirma microarray-based MTC cassette.

**Results:**

The final MTC classifier was blindly tested on 211 preoperative FNAB specimens with subsequent surgical pathology, including 21 MTC and 190 non-MTC specimens from benign and malignant thyroid nodules independent from those used in training. The classifier had 100% sensitivity (21/21 MTC FNAB specimens correctly called positive; 95% confidence interval [CI] = 83.9–100%) and 100% specificity (190/190 non-MTC FNAs correctly called negative; CI = 98.1–100%). All positive samples had pathological confirmation of MTC, while all negative samples were negative for MTC on surgical pathology.

**Conclusions:**

The RNA-sequencing MTC classifier accurately identified MTC from preoperative thyroid nodule FNAB specimens in an independent validation cohort. This identification may facilitate an MTC-specific preoperative evaluation and resulting treatment.

## Introduction

Medullary thyroid carcinoma (MTC) comprises 1–2% of all thyroid cancer cases, yet it is more likely to cause death than the more common types of thyroid cancer.^1,2^ Ten-year disease-specific survival when disease is confined to the thyroid spreads regionally through extrathyroidal extension or cervical lymph node metastases, or with distant disease is 96%, 77%, and 44%, respectively.^2^ Cytopathological evaluation of thyroid fine-needle aspiration biopsy (FNAB) specimens can fail to raise preoperative suspicion of MTC, missing more than one-half of these important malignancies.^3,4^

In a multicenter international study, Essig et al^5^ reported that among 245 surgically confirmed MTC cases, only 44% were diagnosed as MTC by cytology and another 2% as possible MTC.^5^ Sixteen percent were cytologically diagnosed as malignant or suspicious for malignancy, but without the specific suggestion of MTC. Twenty-six percent had cytological diagnoses that approximated Bethesda category III or IV classification. When MTC is not specifically identified preoperatively, these patients are at risk of receiving an insufficient preoperative evaluation and initial thyroid surgery that is not consistent with accepted guidelines.^1^ Surgical treatment discordant with guideline recommendations has been associated with compromised disease-specific survival.^6^

Early MTC detection and treatment are associated with improved patient outcomes. In a national study from Ireland, median survival was 6.3 years, with better outcomes predicted by younger patient age and lower tumor stage.^7^ In another European series of ∼900 MTC patients, calcitonin and carcinoembryonic antigen (CEA) normalized in only 43% of surgical patients,^8^ with independent predictors of survival also pointing to younger patient age and lower tumor stage.^8^ Others have reported that the response to initial surgical therapy is a better predictor of long-term prognosis compared with TNM stage alone,^9^ but earlier disease identification is shared among patients with more favorable surgical responses and lower TNM stages.

Tuttle and Ganly reported that predictors of excellent response to therapy included lower preoperative calcitonin and CEA levels, smaller primary tumor size, less extensive nodal disease, and early stage disease at presentation.^9^ Surgery when disease is confined to the thyroid gland optimizes the chance for cure. Similar to findings from the United States,^2^ a French experience reported the 10-year survival rate was 96% for disease confined to the thyroid, falling to 75% with nodal involvement and 40% with distant metastasis.^10^ While more effective therapies for metastatic disease are clearly needed, optimizing the initial surgical interaction is also important for long-term survival in MTC.

Basal serum calcitonin screening for MTC in thyroid nodule patients is somewhat controversial, as few patients have definitively high values (>100 ng/L), and diagnostic confusion is created among the greater number with marginally abnormal values of 10–100 ng/L.^1,11^ For example, in a type 2 diabetes population with high cardiovascular risk, 10.8% had serum calcitonin values >10 ng/L, and 2.6% had values >20 ng/L.^12^ A better diagnostic test would be both highly sensitive and highly specific. The utility of calcitonin secretagogues toward resolving borderline basal serum calcitonin values is uncertain,^1^ and pentagastrin is largely unavailable.

A microarray-based Afirma MTC classifier was previously developed and validated for use among cytologically Bethesda categories III–VI nodules to specifically identify MTC.^11^ When Afirma migrated to an RNA-sequencing platform,^13^ a new RNA-sequencing-based MTC classifier was developed using machine learning. Here we report the development and clinical validation of this new classifier.

## Materials and Methods

### Feature selection

**FIG. 1.**
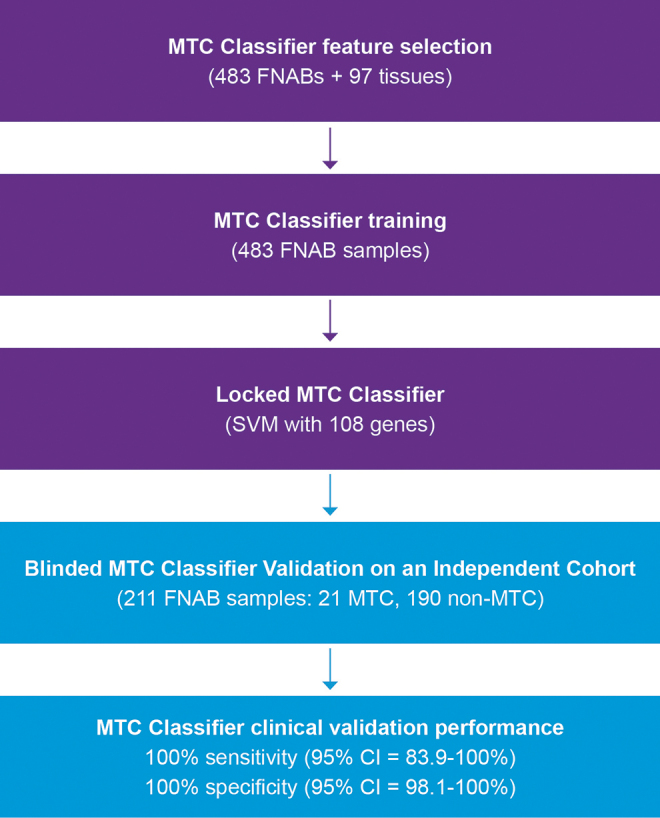
Flow diagram of the RNA-sequencing MTC classifier development and blinded independent validation. MTC, medullary thyroid carcinoma; SVM, support vector machine.

Four hundred eighty-three thyroid nodule FNAB specimens (including 21 from MTC) and 97 independent surgical tissue samples from tumors (including 21 from MTC) were used for feature (gene) selection. The surgical tissues were included to select genes that were differentially expressed (DE) between MTC and non-MTC (neoplasm or mass that was not MTC) for both FNAB and surgical tissue samples to avoid selecting DE genes irrelevant to the MTC phenotype (Fig. 1).

Eight candidate feature sets were constructed using various rules to reduce the number of included DE genes: (candidate feature set 1) DE genes with adjusted *p*-value <1e-6, (candidate feature set 2) DE genes with adjusted *p*-value <1e-6 and log2-fold change >6, (candidate feature sets 3–5) hierarchical clustering on DE genes (adjusted *p*-value <0.01), then select one, 20% or 50% genes from each cluster, (candidate feature sets 6–8) cluster genes by recursive partition using HOPACH^14^ then select 10%, 20%, or 50% genes from each cluster. The 5 genes in the original Afirma microarray-based MTC cassette and 29 literature-derived genes of potential interest were included (Supplementary Table S1) in each of the 8 candidate feature sets.

### Classifier training

**FIG. 2.**
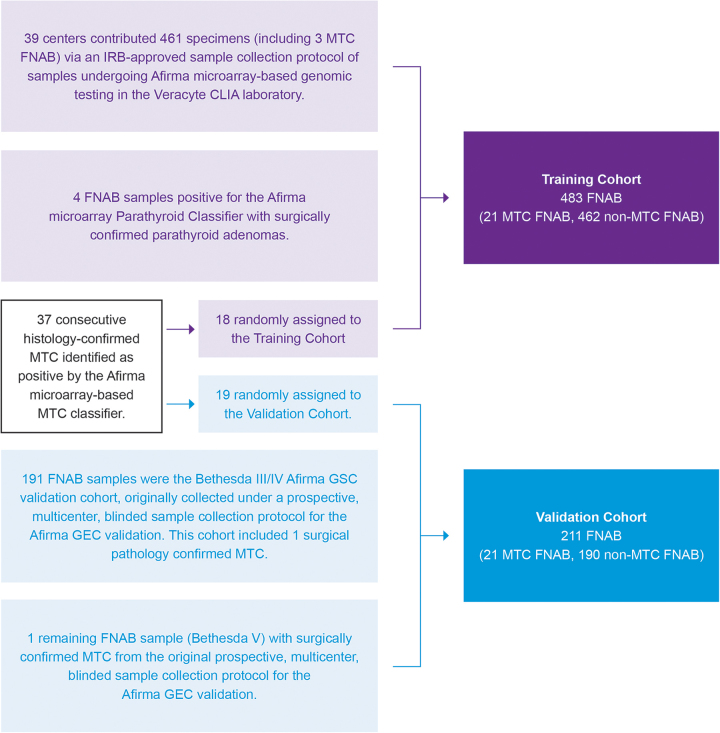
Participant flow diagram. The training cohort was derived from several IRB-approved FNAB sample collection protocols. The protocol for the 461-sample cohort was described in the supplement of Patel et al (see ENHANCE Arm 1, ENHANCE Arm 2, and CLIA-GEC B).^13^ These samples were supplemented with four additional FNAB samples that underwent microarray-based genomic testing in the Veracyte CLIA laboratory and resulted positive by the parathyroid classifier with subsequent surgical confirmation of parathyroid adenoma. The protocol to collect FNAB samples that underwent microarray-based genomic testing in the Veracyte CLIA laboratory and resulted positive by the Medullary Thyroid Cancer Classifier with subsequent surgical confirmation was previously described in Kloos et al.^11^ These samples were randomly assigned here to the training or validation cohorts. The 191 Bethesda categories III/IV samples in the validation cohort here were originally collected under a prospective multicenter blinded sample collection protocol for the Afirma GEC validation as described in Alexander et al.^15^ Collection and attribution of those samples are shown in that publication's Supplementary Figure 1. Those with sufficient residual RNA were then used to validate the Afirma GSC as described in Patel et al,^13^ its Supplementary Figure 2 and are the same samples for this validation. Similarly, the one additional FNAB sample added to this validation cohort was a Bethesda category V sample originally collected and described in the Afirma GEC validation cohort with MTC surgical pathology.^15^ It too met inclusion criteria for the Afirma GSC validation secondary test set.^13^ CLIA, Clinical Laboratory Improvement Amendments; FNAB, fine-needle aspiration biopsy; GEC, Gene Expression Classifier; GSC, Gene Sequencing Classifier.

The 483 thyroid FNAB specimens already mentioned were used for the RNA-sequencing MTC classifier training to generate a result of positive or negative for MTC (Fig. 2). Among the specimens were 21 FNAB specimens from surgically confirmed MTC (5 Bethesda category III, 6 Bethesda category IV, 6 Bethesda category V, 4 Bethesda category VI) and 462 specimens from nodules labeled non-MTC based on surgical and/or molecular evidence. The eight-candidate feature sets already described were tested in both support vector machine (SVM) classifier and logistic regression with elastic net. Fivefold cross-validation was repeated 10 times to better estimate the mean performance of each classifier setting.

### Classifier validation inclusion/exclusion criteria

The validation cohort was an independent (distinct) cohort that did not overlap with samples used for feature selection or classifier training (Fig. 2). Samples were from thyroid FNAB collected from patients 18 years of age and older, with dedicated FNAB passes immediately placed in the Veracyte-provided RNA protective solution tube, chilled shipping (<25°C), stored at −80°C, and contained >15 ng RNA. FNAB samples from only one nodule per patient were included. Samples with inadequate or insufficient RNA were not included in the validation cohort. Such samples are routinely excluded from commercial testing. Only samples with the key study metric of a surgically confirmed pathology diagnosis were included in the validation cohort.

### Validation cohort reference standards

The reference standard for a non-MTC label was a surgical histology diagnosis other than MTC. The reference standard for an MTC label was a surgical histology diagnosis of MTC. Among the 21 MTC FNAB specimens used for validation testing, all had a diagnosis of MTC by surgical pathology. All reference labels were assigned and locked before the development of the Afirma RNA-sequencing MTC classifier.

### Independent classifier validation

The final RNA-sequencing MTC classifier was blindly tested on 211 retrospective FNAB samples that were independent from those used in classifier training and that included 21 MTC (Table 1) and 190 non-MTC samples from benign and malignant thyroid samples. In total, 191 of these samples were the Bethesda categories III/IV Afirma Gene Sequencing Classifier validation cohort that included 1 surgical pathology confirmed MTC (Table 1 sample 1) and 190 surgical pathology confirmed non-MTC samples (surgical histologies listed in Table 5 of Patel et al^13^).

**Table 1.** Demographics of the 21 Medullary Thyroid Carcinoma Nodules in the Clinical Validation Cohort

MTC FNA Sample	Patient age (years)	Sex	Thyroid nodule location	Nodule largest ultrasound dimension (cm)	Cytology Bethesda category	Preoperative basal serum calcitonin (ng/L)	TNM
1	43	Female	Right	2.9	IV	NA	T1bNXMX^a^
2	66	Female	Right	1.5	V	NA	T1bN0MX
3	49	Female	Right	1.0	III	1200	NA
4	18	Female	Left	1.7	V	18	T1bN0MX
5	78	Female	Right	4.0	III	10,702	T2N0MX
6	35	Female	Left	2.5	III	1095	T2N1bMX
7	52	Female	Right	2.1	V	6912	T2N1bMX
8	52	Male	Left	5.1	V	6149	T3aN1bMX
9	62	Female	Left	1.5	III	122	T1bNXMX
10	55	Male	Right	5.3	IV	6866	T3aN0MX
11	73	Female	Right	4.0	III	NA	NA
12	44	Male	Right	0.7	IV	8	T1aN1aMX
13	35	Female	Right	1.0	V	34	T1aN1MX
14	69	Female	Right	1.6	IV	111	T1bNXMX
15	58	Female	Right	NA	III	495	T1bN0MX
16	25	Female	Left	2.1	V	158	T2NXMX
17	49	Female	Left	1.2	III	61	T1aN0MX
18	48	Female	Right	1.7	III	4000	T2N1MX
19	71	Female	Left	0.8	IV	NA	T1aNXMX
20	50	Male	Left	1.0	IV	92	T1aNXMX
21	65	Female	Left	1.4	V	157	T1bN0MX
*Median (range) or percent*	*52 (18–78)*	*81% Female*	*57% Right*	*2.1 (0.7–5.3)*	*38% III* *29% IV* *33% V*	*158 (8–10,702)*	*Stage I: 53%* *Stage II: 16%* *Stage III: 16%* *Stage IVA: 16%*

All had surgical pathology confirmed MTC. Demographics of 190 non-MTC nodules in the clinical validation cohort are reported in the Materials and Methods section. Cancer staging^b^ per AJCC 8th edition.^16^

Summary statistics exclude missing data.

^a^ Tumor size at surgical pathology was 1.8 cm.

^b^ Stage was calculated by assuming that NX and MX are NO and MO, respectively. N1 unspecified (i.e., N1a vs. N1b) was assumed to be N1a.

AJCC, American Joint Committee on Cancer; FNA, fine-needle aspiration; MTC, medullary thyroid carcinoma; NA, calcitonin not available, or accessible medical records are insufficiently detailed for accurate TNM classification.

These 191 samples were originally collected under a prospective multicenter blinded sample collection protocol for the Afirma Gene Expression Classifier (GEC) validation.^15^ The 190 non-MTC FNAB samples came from 182 patients with a mean age of 52 years (range 22–85); 77% were female, with a mean nodule size of 2.6 cm (range 1.0–9.1). The remaining 20 MTC samples included the 1 remaining MTC sample from the Afirma GEC validation cohort^15^ (Table 1 sample 2) and 19 histology-confirmed MTC samples identified as positive by the Afirma microarray-based MTC classifier by June 2013.

### Statistics

Statistical analyses were performed using R statistical software version 3.2.3. The exact binomial test was used to calculate 95% confidence intervals (CIs). One-sample proportion test power analysis was performed using R package pwr. DE analysis was conducted using DESeq2,^17^ and the *p*-values were multiple-hypothesis corrected using the Benjamini–Hochberg procedure.^18^ We evaluated test performance using sensitivity and specificity. For sample size considerations based on a one-sample proportion test power analysis, the null hypothesis of a sensitivity of 90% could be rejected with >90% power at the 0.05 significance level if the classifier could demonstrate 100% sensitivity.

### Institutional review board approval

Specimen collection and research were performed with patient consent or IRB waiver as approved by institution-specific institutional review boards as well as by Liberty IRB (DeLand, Florida; now Chesapeake IRB) and Copernicus Group Independent Review Board (Cary, NC).

## Results

### Classifier selection

All trained classifiers showed 100% sensitivity and specificity on the training set. The following criteria were used to select the single most robust model (classifier): (1) greatest distance between minimum MTC and maximum non-MTC classifier logit scores, (2) smallest logit score variability among controls that were sequenced repeatedly together with training samples, (3) highest logit score correlation with Afirma microarray-based MTC cassette, and (4) smallest number of genes in the model.

A SVM classifier using candidate feature set 2 (gene inclusion required adjusted *p*-value <1e-6 and log2-fold change >6 for both MTC FNAB specimens and tissues compared with those without MTC) was selected as the final model for subsequent independent validation. This locked classifier includes 108 genes (Supplementary Table S1).

### Independent validation

The final RNA-sequencing MTC classifier was blindly tested on 211 independent preoperative FNAB specimens whose surgical pathology included 21 MTC and 190 non-MTC specimens from benign and malignant thyroid samples (Fig. 1). The RNA-sequencing MTC classifier had 100% sensitivity (21/21 MTC FNAB specimens correctly called positive; CI = 83.9–100%) and 100% specificity (190/190 non-MTC FNAB specimens correctly called negative; CI = 98.1–100%).

All positive samples had surgical confirmation of MTC, while all negative samples were negative for MTC on surgical pathology. The age range of patients with MTC was 18–78 years, 81% were female, their largest tumor dimension on ultrasound range was 0.7–5.3 cm, preoperative basal serum calcitonin range was 8–10,702 ng/L, FNAB cytology was Bethesda category III (38%), IV (29%), or V (33%), while 53% were stage I and 47% were stage II and higher (Table 1).

High specificity was achieved by providing correct negative MTC classifier results on all 54 benign follicular nodules/hyperplastic nodules, 54 follicular adenomas, 17 follicular tumors of uncertain malignant potential/well-differentiated tumors of uncertain malignant potential, 17 oncocytic (Hürthle cell) adenomas, 2 chronic lymphocytic (Hashimoto) thyroiditis, 1 hyalinizing trabecular tumor, 17 papillary thyroid carcinoma (including papillary thyroid carcinoma tall cell variant), 11 papillary thyroid carcinoma follicular variant, 9 oncocytic (Hürthle cell) carcinomas, 7 follicular carcinomas or well-differentiated carcinoma not otherwise specified, and 1 poorly differentiated thyroid carcinoma.

## Discussion

In this study we report the development and independently blinded clinical validation of the Afirma RNA-sequencing MTC classifier that has demonstrated high sensitivity and specificity. All positive samples had surgical confirmation of MTC, while all negative samples were negative for MTC on surgical pathology. Throughout training and validation, highly accurate performance was seen among cytologically Bethesda categories III–VI FNA samples.

All MTCs were correctly identified in both cross-validation of the training cohort and the independent validation cohort. MTCs in the training cohort were 24% Bethesda category III, 29% Bethesda category IV, 29% Bethesda category V, and 19% Bethesda category VI. MTCs in the validation cohort were 38% Bethesda category III, 29% Bethesda category IV, and 33% Bethesda category V. The MTC classifier correctly identified MTCs as small as 7 mm and in patients as young as 18 years old, even when baseline serum calcitonin was <20 ng/L (Table 1).

Separate from the clinical validation performance reported here, analytical validation of the Afirma RNA-sequencing MTC classifier was previously published.^19^ This included measures of accuracy between different laboratories, precision, analytical sensitivity, and analytical specificity. The positive signal in an MTC sample was shown to tolerate up to 75% dilution by benign RNA and still yield a positive MTC classifier result. Beyond the analytical and clinical validation data, future investigations should evaluate cost-effectiveness.

The indication for Afirma RNA-sequencing MTC classifier consideration is among cytologically indeterminate (Bethesda categories III/IV), suspicious (Bethesda category V), or malignant (Bethesda category VI) thyroid nodules that lack a definitive diagnosis of MTC when such a diagnosis would alter their treatment. While MTC is not typically suspected among FNAB samples read as cytologically indeterminate, so too can MTC be identified among cytologically malignant samples that are not specifically identified as MTC.^3,5,11,20^ Conversely, some FNAB specimens cytologically suspected of MTC are found to have alternative diagnoses upon surgical resection.^11^

Without the specific identification of MTC, MTC patients are unlikely to receive the appropriate evaluation, testing, staging, and treatment that they would otherwise receive according to management guidelines.^1,21^ The preoperative evaluation includes germline *RET* mutation testing and consideration and treatment of MEN2-associated pheochromocytoma if present.^1^ Thyroid surgery on an MEN2 patient with an untreated pheochromocytoma can result in perioperative morbidity and death.^22^ The minimal thyroid surgery recommended for MTC that presents as a thyroid nodule (≥1 cm) is total thyroidectomy and prophylactic central neck dissection.^1,21^ This is a more extensive surgery than is recommended for cytologically indeterminate thyroid nodules or most differentiated thyroid carcinomas that are confined to the thyroid.^23^

Failure to diagnose MTC preoperatively may result in delayed and insufficient initial treatment and lead to subsequent surgeries. This may partly explain why 40% or more of initial surgical treatments for MTC patients are less extensive than advised by guideline recommendations.^6,24,25^ Kuo et al^25^ reported that lymph node dissection was associated with decreased MTC recurrence leading to reoperation (hazard ratio, 0.53; CI = 0.30–0.93) according to data from the California Cancer Registry and the Office of Statewide Health Planning and Development.^25^

Panigrahi et al analyzed Surveillance, Epidemiology, and End Results (SEER) data and reported that disease-specific survival was shorter in patients who did not receive appropriate surgery according to guidelines.^6^ Randle et al reported improved surgical approach and survival in the most recent decade according to SEER data, yet nearly one-quarter of MTC patients remain insufficiently treated.^2^ They speculated that limitations in the preoperative diagnosis of MTC by FNAB-mediated cytology evaluation may have contributed to this treatment insufficiency. It is possible that stronger clinical practice guideline recognition and endorsement of preoperative MTC molecular diagnostic testing among Bethesda categories III–VI nodules may improve patient outcomes.

A limitation of this study is the relatively small number of MTC cases included in the cross-validation cohort and the independent and blinded validation cohort. MTC has a low incidence among thyroid cancers and is rare by incidence in the general population,^2^ which makes assembling large cohorts of preoperative FNAB specimens from them difficult. Still, the number of MTCs included here is the largest independent validation cohort among available molecular diagnostic tests that specifically identify MTC. In the genomic space, the molecular patten of MTC is very distinct from non-MTC samples. Given this, it seems unlikely that a greater number of MTC samples in a validation cohort would significantly alter test performance.

In real-world clinical experience, publications with Afirma RNA-sequencing MTC classifier testing conducted independently from Veracyte, there have been no false negative or false positive results reported among >2100 thyroid nodules.^26–39^ This robust finding may also mitigate the potential limitation of some authors having a multiplicity of interests. Statistical analyses were performed by Veracyte. Reference diagnoses were assigned by surgical pathologists not employed by Veracyte. Most authors have no Veracyte financial interests. Another limitation of this study is the lack of Non-Invasive Follicular Thyroid neoplasm with Papillary-like nuclear features (NIFTP) in the validation cohort.

This tumor type nomenclature came into existence only after this non-MTC validation cohort was collected and their surgical pathology diagnoses assigned.^13,15^ If any NIFTP exist in this cohort, they resulted negative with the Afirma RNA-sequencing MTC classifier. Only 26 of the 42 MTC cases with FNAB samples used here in training or validation have known germline *RET* protooncogene results available.

Three had germline RET mutations, but none were present in the validation cohort. In this study, we did not investigate the FNAB samples for gene point mutations or fusions. We previously reported those findings from RNA sequencing of a consecutive cohort of 152 Afirma RNA-sequencing MTC classifier positive cases: 70% had at least 1 alteration identified, the majority being point alterations of *RET*.^40^ The MTC classifier performance was not studied in conjunction with calcitonin measurements in this study.

## Conclusion

We report the development and blinded independent clinical validation of the Afirma RNA-sequencing MTC classifier. All positive samples had surgical pathology confirmation of MTC, while all negative samples were negative for MTC postoperatively. This test facilitates the preoperative diagnosis of MTC to enable appropriate initial treatment of MTC patients with Bethesda categories III–VI FNA cytology.

## Supplementary Material

Supplemental data
