# Supplementary material for: Preoperative Identification of Medullary Thyroid Carcinoma (MTC): Clinical Validation of the Afirma MTC RNA-Sequencing Classifier
Source: Thyroid. 2022 Sep 14;32(9):1069–76. doi: 10.1089/thy.2022.0189 (PMC9526471; doi:10.1089/thy.2022.0189)
Supplement: Supplemental data [file Suppl_TableS1.docx]

Supplementary Table 1. The five genes in the original Afirma microarray-based MTC cassette and 29 literature-derived genes of potential interest were included in each of the eight candidate classifiers. The additional 74 genes were selected based on the feature section process described in the text that led to the final Afirma RNA-Sequencing MTC classifier (108 genes total).

| **Gene name** | **Reason for inclusion** |
| --- | --- |
| CEACAM5 | Afirma microarray-based MTC Cassette |
| CALCA | Afirma microarray-based MTC Cassette |
| SCG3 | Afirma microarray-based MTC Cassette |
| SYT4 | Afirma microarray-based MTC Cassette |
| SCN9A | Afirma microarray-based MTC Cassette |
| CEACAM7 | Literature-derived gene of potential interest |
| CEACAM6 | Literature-derived gene of potential interest |
| CEACAM19 | Literature-derived gene of potential interest |
| CEACAM21 | Literature-derived gene of potential interest |
| KRT18 | Literature-derived gene of potential interest |
| KRT8 | Literature-derived gene of potential interest |
| CEACAM16 | Literature-derived gene of potential interest |
| CEACAM3 | Literature-derived gene of potential interest |
| CEACAM18 | Literature-derived gene of potential interest |
| CEACAM4 | Literature-derived gene of potential interest |
| CEACAM1 | Literature-derived gene of potential interest |
| KRT20 | Literature-derived gene of potential interest |
| CEACAM20 | Literature-derived gene of potential interest |
| KRT9 | Literature-derived gene of potential interest |
| KRT12 | Literature-derived gene of potential interest |
| CEACAM8 | Literature-derived gene of potential interest |
| KRT1 | Literature-derived gene of potential interest |
| KRT10 | Literature-derived gene of potential interest |
| KRT2 | Literature-derived gene of potential interest |
| KRT4 | Literature-derived gene of potential interest |
| KRT17 | Literature-derived gene of potential interest |
| KRT7 | Literature-derived gene of potential interest |
| KRT19 | Literature-derived gene of potential interest |
| KRT3 | Literature-derived gene of potential interest |
| KRT14 | Literature-derived gene of potential interest |
| KRT13 | Literature-derived gene of potential interest |
| KRT16 | Literature-derived gene of potential interest |
| TTF1 | Literature-derived gene of potential interest |
| KRT5 | Literature-derived gene of potential interest |
| NEFM | Feature selection |
| NSG2 | Feature selection |
| SYT1 | Feature selection |
| SLC18A1 | Feature selection |
| ST18 | Feature selection |
| VGF | Feature selection |
| USP41 | Feature selection |
| HOXB5 | Feature selection |
| ADCYAP1 | Feature selection |
| DCX | Feature selection |
| OPRD1 | Feature selection |
| PHGR1 | Feature selection |
| PTPRN2 | Feature selection |
| ANO4 | Feature selection |
| GRP | Feature selection |
| INSM1 | Feature selection |
| ASCL1 | Feature selection |
| ST8SIA3 | Feature selection |
| RP11-672L10.2 | Feature selection |
| CACNG2 | Feature selection |
| SCGN | Feature selection |
| CABP7 | Feature selection |
| SRRM4 | Feature selection |
| SNAP25 | Feature selection |
| HTR5A | Feature selection |
| AMER3 | Feature selection |
| RAB3C | Feature selection |
| SCG2 | Feature selection |
| ELAVL4 | Feature selection |
| CALY | Feature selection |
| GFRA4 | Feature selection |
| TNR | Feature selection |
| OTOP3 | Feature selection |
| CHGB | Feature selection |
| MIR7-3HG | Feature selection |
| SVOP | Feature selection |
| SYN1 | Feature selection |
| PTPRN | Feature selection |
| CHRNB2 | Feature selection |
| TAGLN3 | Feature selection |
| DRD2 | Feature selection |
| UNC13A | Feature selection |
| DDC | Feature selection |
| CELF3 | Feature selection |
| SYT2 | Feature selection |
| PON1 | Feature selection |
| GAP43 | Feature selection |
| KCNK9 | Feature selection |
| MRAP2 | Feature selection |
| SEZ6 | Feature selection |
| PCSK1 | Feature selection |
| PPFIA2 | Feature selection |
| NRXN1 | Feature selection |
| CHRNA3 | Feature selection |
| SLC8A2 | Feature selection |
| KIRREL2 | Feature selection |
| CHST9 | Feature selection |
| MEP1B | Feature selection |
| NKAIN2 | Feature selection |
| RXFP2 | Feature selection |
| FGF14 | Feature selection |
| BPIFB2 | Feature selection |
| NPTX1 | Feature selection |
| FSTL5 | Feature selection |
| GRIA2 | Feature selection |
| GABRR1 | Feature selection |
| CAPN8 | Feature selection |
| KCNF1 | Feature selection |
| UCN3 | Feature selection |
| RALYL | Feature selection |
| GP2 | Feature selection |
| TAAR1 | Feature selection |
| STMN2 | Feature selection |
| SIM1 | Feature selection |
